# MiR-15b Targets Cyclin D1 to Regulate Proliferation and Apoptosis in Glioma Cells

**DOI:** 10.1155/2014/687826

**Published:** 2014-06-04

**Authors:** Guan Sun, Lei Shi, Shushan Yan, Zhengqiang Wan, Nan Jiang, Linshan Fu, Min Li, Jun Guo

**Affiliations:** ^1^Department of Neurosurgery, Fourth Affiliated Hospital of Nantong University, First Hospital of Yancheng, Yancheng 224001, China; ^2^Department of Neurosurgery, The First People's Hospital of Kunshan Affiliated with Jiangsu University, Suzhou 215300, China; ^3^Department of Medical Oncology, The Eighty-First Hospital of People's Liberation Army, Nanjing 210002, China; ^4^Department of Neurosurgery, Jiangning Hospital Affiliated with Nanjing Medical University, Nanjing 211100, China

## Abstract

*Aim*. To investigate the role and mechanism of miR-15b in the proliferation and apoptosis of glioma. *Methods*. The miR-15b mimics were transfected into human glioma cells to upregulate the miR-15b expression. Cyclin D1 was determined by both western blotting analysis and luciferase reporter assay. Methylthiazol tetrazolium (MTT) and flow cytometry were employed to detect the cell proliferation, cell cycle, and apoptosis. *Results*. Overexpression of miR-15b inhibits proliferation by arrested cell cycle progression and induces apoptosis, possibly by directly targeting Cyclin D1. Both luciferase assay and bioinformatics search revealed a putative target site of miR-15b binding to the 3′-UTR of Cyclin D1. Moreover, expression of miR-15b in glioma tissues was found to be inversely correlated with Cyclin D1 expression. Enforced Cyclin D1 could abrogate the miR-15b-mediated cell cycle arrest and apoptosis. *Conclusions*. Our findings identified that miR-15b may function as a glioma suppressor by targeting the Cyclin D1, which may provide a novel therapeutic strategy for treatment of glioma.

## 1. Background

Glioma represents the majority of primary malignant brain tumors with low survival rate [1]. Despite advance in surgery, radiotherapy, and chemotherapy, over 50% of patients die within one year by diagnosis of glioblastoma [2]. Thus, it is very urgent to explore the mechanisms that underlie the occurrence and development of glioma.

In the past few years, miRNAs considered as glioma biological targets were widely studied. To our knowledge, a growing body of evidence indicates that miRNAs play fundamental roles in the tumorigenesis of human cancer including glioma, affecting cell survival, proliferation, differentiation, metabolism, angiogenesis, and stem cell generation [3, 4]. More and more numbers of miRNAs including miR-21, miR-125b, let-7, miR-9, and miR-137, as tumor suppressors or oncogenes by negatively regulating oncogenes or tumor-suppressor genes, have been verified to expose aberrant expression in glioma [5–9]. One given miRNA is supposed to have multiple gene targets while one given target also may have simultaneous multiple upstream miRNAs.

For example, we have recently reported that forced expression of miR-137 suppresses proliferation and invasiveness in cultured glioma cells by directly targeting Rac1. Till now, it has been verified that miR-137 directly regulates a wide variety of genes such as CDC42, CDK6, KIT, and AEG-1 in several cancer tissues [10–12]. MiR-34a negatively regulates surviving protein expression and inhibits gastric cancer cell proliferation and invasion [13]. Thus, altered expression miRNAs and their simultaneous target genes have been demonstrated to contribute to the development of various cancers.

Recently, miR-15b has been declared to be involved in progression of an element of cancer tissues. Ectopic miR-15b induces apoptosis by targeting BCL2, sensitized SGC7901/VCR cells to VCR-induced apoptosis [14]. MiR-15b directly targeted FUT2 and then increased levels of Globo H to enhance HCC cell proliferation [15]. However, the molecular biological effects and regulatory mechanism of miR-15b in glioma cells remain elusive.

Here, we investigated the effects of miR-15b in glioma cells and explored the detailed mechanisms involved. Our data revealed that miR-15b was substantially decreased in glioma specimens compared to the matched normal brain tissues. Further studies showed that Cyclin D1 was a novel direct target of miR-15b and was frequently downregulated in glioma tissues and cell lines. MiR-15b inhibited U87 and LN229 glioma cells growth at least partially by regulating the 3′-untranslated regions (3′-UTR) of Cyclin D1. In consequence, miR-15b may act as the role of tumor suppressor in glioma cells.

## 2. Materials and Methods

### 2.1. Human Tissue Samples and Cell Lines

Human U87 and LN229 glioma cell lines were purchased from the Chinese Academy of Sciences Cell Bank. All cells were maintained in a 37°C, 5% CO_2_ incubator in Dulbecco's modified eagle's medium (DMEM) (Gibco, USA) supplemented with 10% fetal bovine serum (Invitrogen).

### 2.2. Oligonucleotides and Cell Transfection

The oligonucleotides were chemically synthesized by GenePharma (Shanghai, China) bases with the following sequences: hsa-miR-15b mimics (sense: 5′-UAGCAGCACAUCAUGGUUUACA-3′, antisense: 5′-UAAACAUGAUGUGCUGCUGUU-3′) and scrambled miR-15b (sense: 5′-UUCUCCGAACGUGUCACGUTT-3′, anti-sense: 5′-ACGUGACACGUUCGGAGAATT-3′). For the Cyclin D1 plasmid vectors (Genesil, Wuhan, China) were constructed by Wuhan Genesil. Vectors were transfected into human glioma cell lines with FuGENE HD6 (Roche, Basel, Switzerland) according to the manufacturer's instructions and screened by the aminoglycoside G418. For transfection, 2 × 10^5^ cells were placed into each well of six well plates for 12 hours. Mimics were allowed to form transfection complexes with Lipofectamine 2000 (Invitrogen) according to the manufacturer's instructions (Invitrogen, Carlsbad, USA), subsequently added to U87 and U229 glioma cells, and left to incubate for 8 h before medium change. Experiments were divided into three groups as blank control group (blank), miRNA scrambled group (scramble), and hsa-miR-15b mimics group (miR-15b mimics).

### 2.3. Quantitative Real-Time PCR (qRT-PCR) Analysis of MiR-15b and Cyclin D1

Total RNA was extracted from clinical tissues and transfected cells, using Trizol reagent (Invitrogen, USA). The ABI 7300 HT sequence detection system (Applied Biosystems, Foster City, CA) was used for Taqman-based real-time reverse transcription-polymerase chain reaction (RT-PCR) assays to detect the relative levels of miR-15b and Cyclin D1 in glioma samples and transfected cells. Primers and probes of the miR-15b and Cyclin D1 for Taqman miRNA assays were purchased from Applied Biosystems. The quantitative miR-15b and Cyclin D1 expression date was calculated by using a 2^−^ΔΔCt method.

### 2.4. MTT Assay

Cells in the log phase of growth were seeded into 96-well plates at 3 × 10^3^ cells per well. Subsequently, 50 *μ*L of MTT dilution (5 mg/mL, KeyGEN, China) was added into each well at each day of the consecutive 3 days after transfection and the cells were incubated at 37°C for additional 4 h. The supernatant was discarded and 200 *μ*L of DMSO was added to each well to dissolve the precipitate. Optical density (OD) was measured at the wavelength of 570 nm and data were presented as the mean ± SD.

### 2.5. Cell Cycle Assay

Cells were washed with PBS and fixed with 70% ethanol for at least 1 h. After extensive washing, the cells were suspended in HBSS (Hank's balanced salt solution) containing 50 *μ*g/mL PI and 50 *μ*g/mL RNase A and incubated for 1 h at room temperature and analyzed by FACScan (Becton Dickinson, USA). Cell cycle analysis was analyzed by ModFit software. Experiments were performed in triplicate. Results were presented as % of cell in a particular phase.

### 2.6. Apoptosis Assay

1 × 10^6^ cells were plated into 6-well plates, and, after transfection with each oligonucleotide for 48 h, the Annexin V FITC and PI double stain were used to detect and quantify apoptosis by flow cytometry.

### 2.7. Luciferase Reporter Assay

The full length 3′UTR region of the Cyclin D1 gene was subcloned into luciferase reporter vectors from human genomic DNA and the mutant Cyclin D1 vectors were constructed. Cells of 60–70% confluence in 24-well plates were cotransfected with luciferase reporter vectors and miR-15b expressing vectors, and a 1ng pRLSV40 Renilla luciferase construct was used for normalization. After 48 h, luciferase activity was analyzed by the dual-luciferase reporter assay system according to the manufacturer's protocols (Promega, Madison, USA).

### 2.8. Western Blotting Analysis

At 48 h after transfection with miR-15b mimics or scramble oligonucleotides, total proteins from control and transfected cells were extracted and the protein concentration was determined by BSA method (keyGEN, China). 30 *μ*g of protein lysates was subjected to SDS-PAGE in 10% acrylamide gel each sample. Then, the electrophoresed proteins were transferred to NC membranes (Millipore Corporation, USA). After that, the membrane was blocked in 5% nonfat milk and incubated with diluted antibodies against Cyclin D1 (1 : 200, Santa Cruz, USA) overnight at 4°C, followed by incubation with HRP-conjugated secondary antibody (1 : 2500, Santa Cruz, USA). After washing with stripping buffer, the membrane was reprobed with GAPDH (1 : 5000, Kangchen, China), using ultra enhanced chemiluminescence western blotting detection reagents. All Western bands were quantified by densitometry and are presented in the form of a bar graph.

### 2.9. Statistics Analysis

Data were analyzed with SPSS 13.0 statistical evaluation for data analysis that was determined by *t* test, one-way ANOVA, and Pearson's correlation analysis. Differences with *P* < 0.05 were considered statistically significant.

## 3. Results

### 3.1. MiR-15b Expression in Glioma Tissues

**Figure 1:**
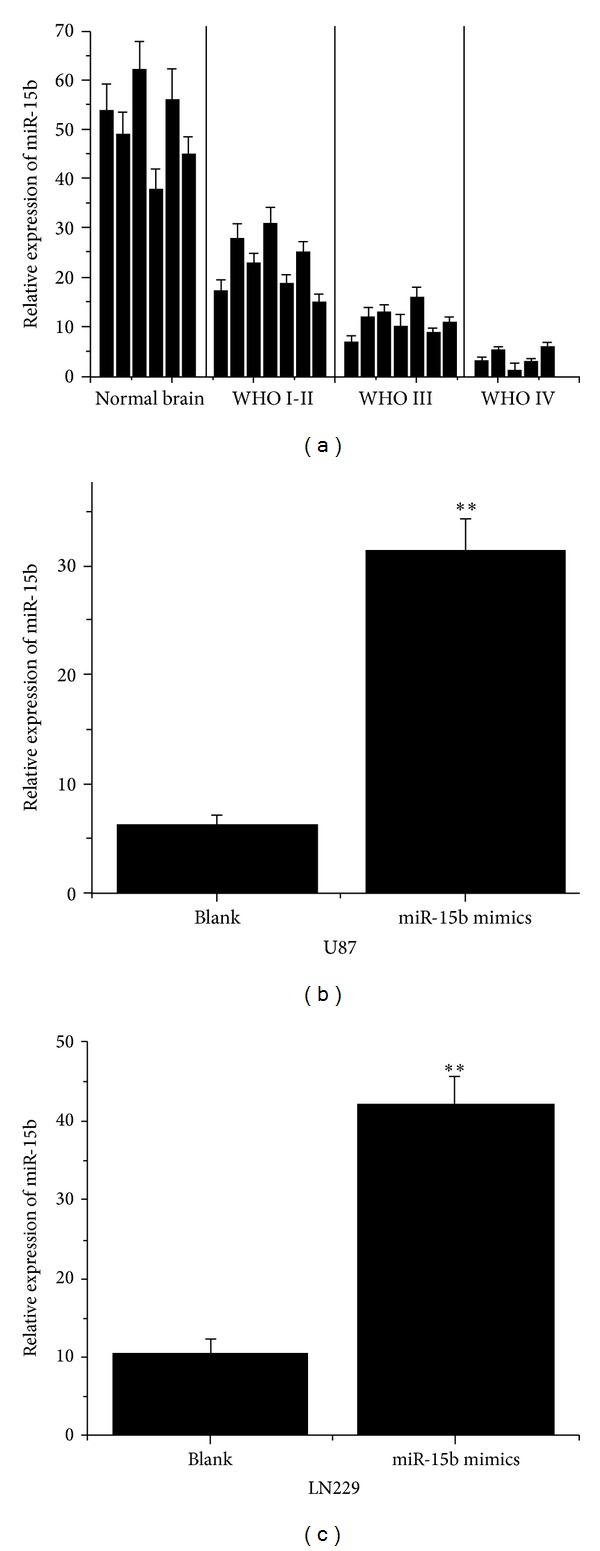
(a) MiR-15b expression in glioma tissues and normal brain specimens. The relative levels of miR-15b were measured by real-time PCR assay. (b, c) MiR-15b expression increased about 5.02-fold and 3.98-fold, at 48 h after transfection of miR-15b mimics in U87 and LN229 cells, respectively. Total RNA was extracted using Trizol reagent. The relative expression of miR-15b was calculated by using a 2^−^ΔΔCt method. The data are presented as the mean ± SD. **P < 0.05 compared to the control.

To explore the expression of miR-15b in glioma tissues, 7 I-II, 7 III, and 5 IV grades and 6 normal brain tissues were examined by real-time PCR assay. As shown in Figure 1, miR-15b expression decreased markedly in glioma specimens in comparison to the normal brain tissues and showed an obviously downward trend with ascending tumor pathological grades (Figure 1(a)). Similarly, lower expression of miR-15b was observed in U87 and L229 glioma cells. To determine the biological functions of miR-15b in glioma cells, we used chemically synthesized modified oligonucleotides to transfect into U87 and L229 cell lines. The results of real-time PCR assay suggested that miR-15b was elevated effectively after transfection in glioma cell lines (Figures 1(b) and 1(c)).

### 3.2. Cyclin D1 Is a Direct Target of MiR-15b

**Figure 2:**
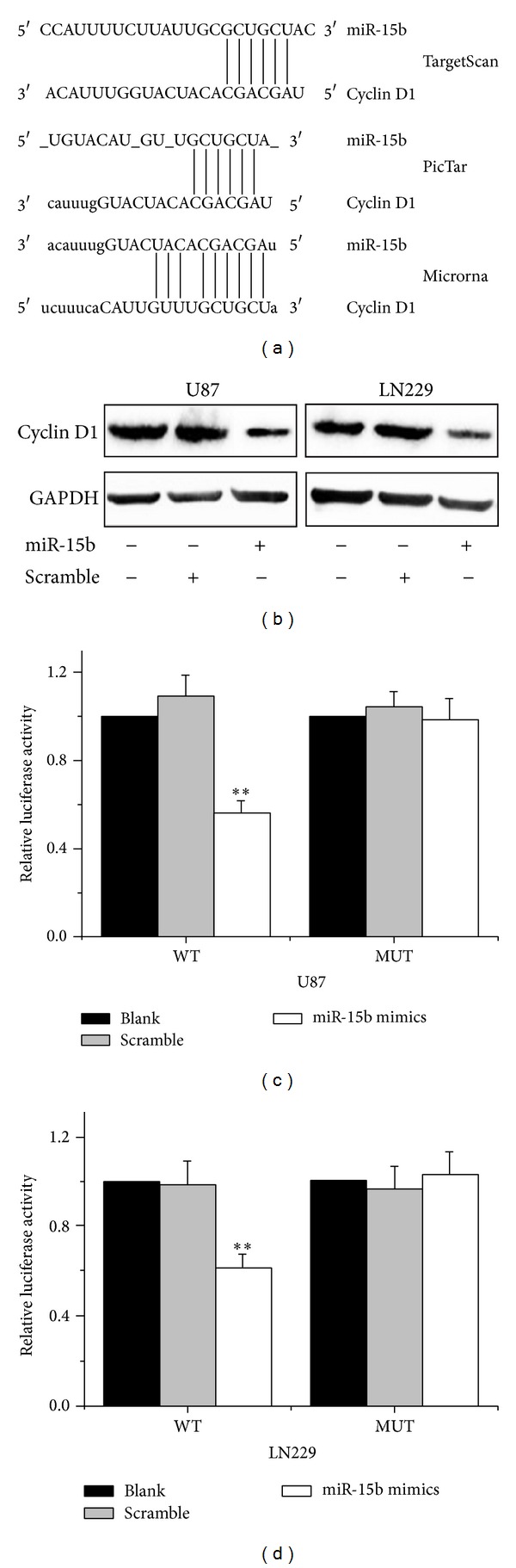
Cyclin D1 is a direct target of miR-15b in glioma cells. (a) Bioinformatics analysis of the predicted interactions of miR-15b with the binding sequence at the 3′UTR of Cyclin D1 mRNA. (b) Overexpression of miR15b downregulates endogenous Cyclin D1 expression level in U87 and LN229 glioma cells. (c, d) Luciferase assay showed a reduction of luciferase expression following the cotransfection of pGL3-Cyclin D1 vector together with miR-15b. The data were normalized by the ratio of firefly and Renilla luciferase activities. The data are presented as the mean ± SD. **P < 0.05 compared to the control.

Three bioinformatic algorithms (TargetScan, PicTar, and miRanda) were employed to identify a large number of potential target genes of miR-15b. Among these candidates, Cyclin D1 was selected for further analysis. Binding sites of miR-15b were observed in the 3′-UTR of Cyclin D1 mRNA; we hypothesized that Cyclin D1 may be a direct target of miR-15b (Figure 2(a)). Western blotting analysis showed that miR-15b could reduce the expression of Cyclin D1 in both U87 and LN229 cells (Figure 2(b)). To further confirm whether Cyclin D1 is a direct target of miR-15b, a reporter plasmid harboring the wild-type 3′-UTR region of Cyclin D1 downstream of the luciferase coding region was constructed. The assay denoted that the overexpression of miR-15b induced an obvious decrease in the luciferase activity of the pGL3-WT Cyclin D1 in both U87 and LN229 cells, indicating that miR-15b directly regulates Cyclin D1 gene by binding to 3′UTR region (Figures 2(c) and 2(d)). These findings suggested that miR-15b directly regulates Cyclin D1 via binding the 3′-UTR of Cyclin D1.

### 3.3. Negative Link between MiR-15b and Cyclin D1 Expression in Glioma Tissues

**Figure 3:**
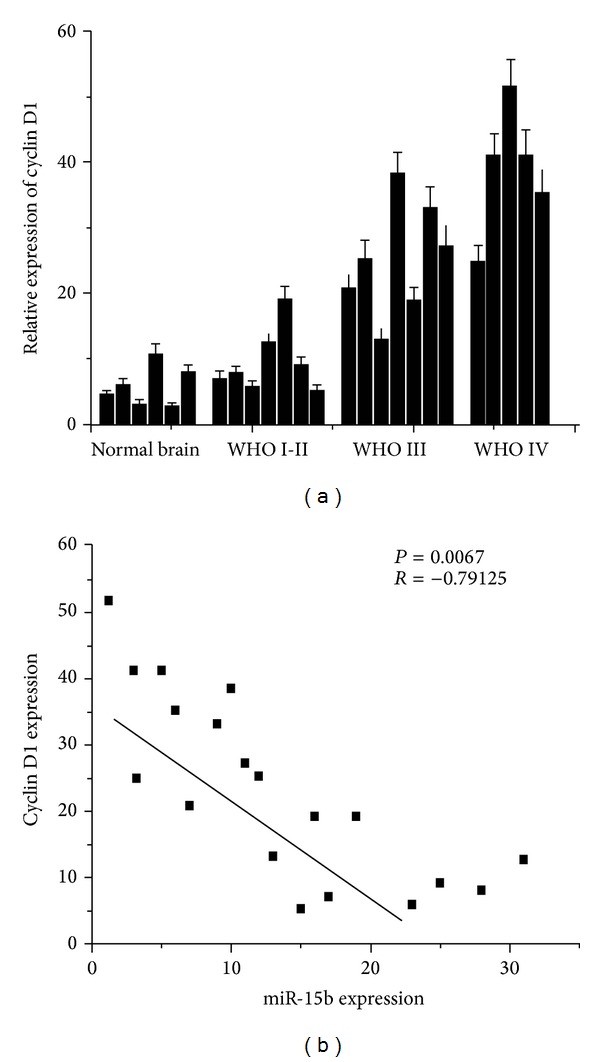
Negative link between miR-15b and Cyclin D1 expression in glioma tissues. (a) The relative expression of Cyclin D1 was measured by real-time PCR assay. (b) Inverse correlation of miR-15b expression with Cyclin D1 mRNA expression using Pearson's correlation analysis.

To investigate the association between miR-15b and Cyclin D1 expression in glioma, we analyzed Cyclin D1 expression by real-time PCR. The higher expression of Cyclin D1 was found in glioma tissues compared to the normal brain tissues. In addition, Pearson's correlation coefficient showed a significant inverse correlation between miR-15b and Cyclin D1 in glioma tissues (*R* = −0.79125  *P* < 0.01) (Figure 3(b)). These results indicate a negative link miR-15b and Cyclin D1 and further confirm that Cyclin D1 is a direct target of miR-15b.

### 3.4. MiR-15b Suppresses the Proliferation of U87 and L229 Glioma Cells In Vitro

**Figure 4:**
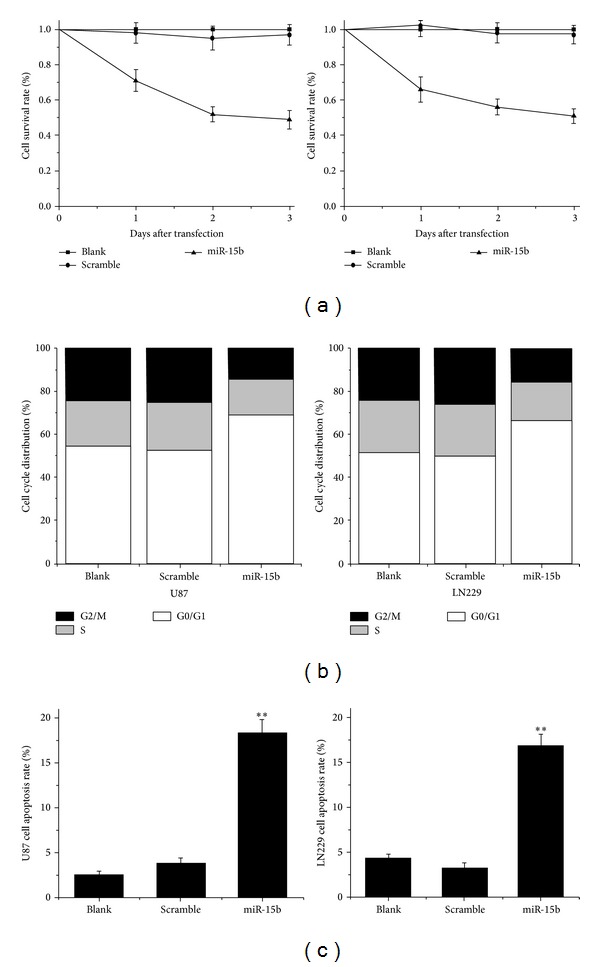
MiR-15b suppresses the growth of glioma cells. (a) MTT assay reveals a significantly inhibitory effect of miR-15b mimics treated cell (Student's *t*-test). (b) Overexpression of miR-15b results in the cell cycle arrest at G0/G1 phases in glioma cells. (c) MiR-15b induced apoptosis in both U87 and L229 glioma cells (Student's *t*-test). The data are presented as the mean ± SD. **P < 0.05 compared to the control.

To determine the effects of miR-15b on proliferation of glioma cells, MTT assay was employed to evaluate the cells growth viability. MiR-15b treated U87 cells showed a significant decrease in proliferation relative to both blank and scramble-treated groups. About 71.17 ± 6.15%, 52.63 ± 4.18%, and 49.49 ± 5.24% survival rates in 1 d, 2 d, and 3 d after transfected time point were shown, respectively, and the similar inhibitory effects were found in LN229 cell (Figure 4(a)). The assay revealed that ectopic expression of miR-15b significantly suppressed the proliferation of glioma cells.

### 3.5. MiR-15b Results in an Increase of Cell Populations in G0/G1 Phase

The cell cycle distribution by flow cytometry assay was employed to explore why miR-15b inhibits glioma cells. As showed in Figure 4(b), the G1/G0 phase fraction of the control and scramble groups was 54.42% and 52.72% while the miR-15b mimics group increased to 69.13% in U87 cells. In the meanwhile, the G1/G0 phase fraction of the control and scramble groups was 51.84 and 49.56% while the miR-15b mimics group increased to 66.32% in LN229 cells. These data suggest that miR-15b mimics lead to the arrest of the cells at G1/G0 phases and delay the progression of cell cycle.

### 3.6. MiR-15b Induced Apoptosis in U87 and L229 Glioma Cells

To investigate the effects of miR-15b on glioma cell apoptosis, we used miR-15b mimics to transfect into U87 and LN229 glioma cells. At 48 h after transfection, apoptosis was measured by flow cytometry. The higher apoptotic rates were found among miR-15b mimics treated U87 cells (18.42 ± 1.46) in comparison to the untreated cells. MiR-15b mimics treated LN229 cells also showed considerable apoptotic cells compared to the untreated cells (Figure 4(c)). The assay implied that miR-15b could induce apoptosis in U87 and L229 glioma cells.

### 3.7. Overexpression of Cyclin D1 Partly Represses the MiR-15b Induced Cell Cycle Arrest in Glioma Cells

**Figure 5:**
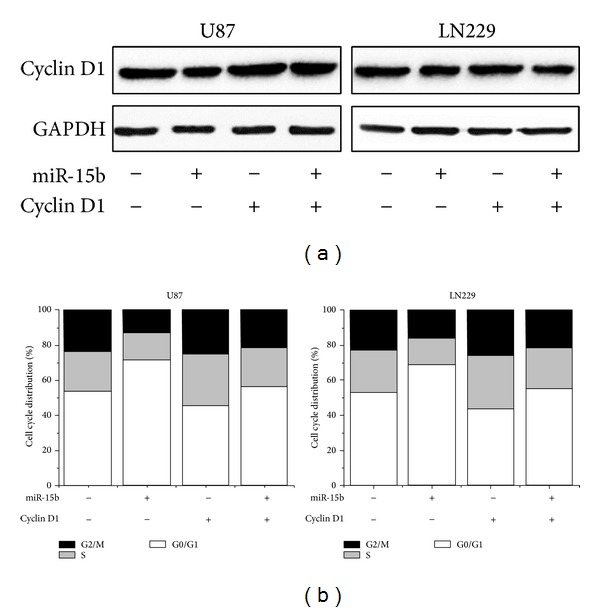
Cyclin D1 plays a crucial role in the suppressive proliferative process of miR-15b in glioma cells. (a) Western bolt analysis displays that ectopic of Cyclin D1 abrogates the miR-15b-mediated Cyclin D1 expression partly. (b) Ectopic expression of Cyclin D1 counteracts the G1 arrest induced by miR-15b in glioma cells. The representative results out of three times of experiments were shown here.

To explore the importance of Cyclin D1 in the process of miR-15b-mediated cell cycle and apoptosis, the plasmid mediated overexpression of Cyclin D1 was transfected into glioma cells accompanying miR-15b mimics. As showed in Figure 5(a), western bolt assay significantly showed that enforced expression of Cyclin D1 at least partly repressed the miR-15b-mediated Cyclin D1 expression in both U87 and LN229 cells. Furthermore, ectopic expression of Cyclin D1 statistically counteracted the G1 arrest induced by miR-15b in glioma cells (Figure 5(b)). Likewise, the miR15b-induced cell apoptosis was rescued considerably. Taken together, the results of this rescue experiment demonstrate that reintroduction of Cyclin D1 could abrogate the inhibitory effects of miR-15b-mediated cell cycle arrest and apoptosis, suggesting that Cyclin D1 is a major target of miR-15b involved in the malignant progression of miR-15b.

## 4. Discussion

MiRNAs are a novel class of small nonprotein coding single-stranded RNA molecules, which are essentially posttranscriptional regulators of gene expression. Increasing studies in recent years have confirmed that miRNAs could be involved in a wide range of biological functions in various human cancers. MiRNAs may function as oncogenes or antioncogenes, leading to aberrant cell proliferation and survival by regulating downstream target genes relevant to signal cascades. Thus, it is important to depict the function and mechanism of miRNAs involved in the occurrence and development of glioma.

MiR-15b was firstly reported by Xi et al. The panel found that it was significantly overexpressed in colorectal cancer compared to normal colorectal sample [16]. Wang et al. revealed that miR-15b was upregulated by using miRNA array analyses for age-matched normal cervix and cervical cancer tissues in combination with northern blot verification [17]. In contrast, a number of studies have shown that miR-15b statistically reduced in tumor tissues, such as gastric cancer and hepatocellular carcinoma [14, 18, 19]. From above discussion, miR-15b expression was different from diverse organic tissues. Thus, we measured the levels of miR-15b in 19 glioma tissues and 6 normal brain tissues. Our data showed that miR-15b was frequently downregulated in glioma tissue as well as glioma cell lines. Furthermore, miR-15b expression was markedly decreased with ascending glioma malignancy, suggesting that miR-15b exists as an antitumor factor in glioma. Although there have been abundant studies reporting that miR-15b may serve as an essential role in the development and progression of human tumors, the effect of miR-15b in mediating glioma cell growth remains unexplored. To explore the potential molecular mechanism of miR-15b functions in glioma cell lines, we transfected miR-15b into U87 and LN229 cells and found that overexpression of miR-15b in vitro statistically inhibited the proliferation of U87 and LN229 glioma cells, arrested miR-15b mimics treated cells in G0/G1 phase, and induced apoptosis in glioma cells.

Cyclin D1 is one of the crucial mitogen cell cycle regulators, which play a pivotal role in the transition of cell cycle from G1 to S phase by binding its partners cyclin dependent kinase 4/6 to form an active protein kinase [20, 21]. Cyclin D1 is frequently overexpressed in a large number of cancers, including colorectal, gastric cancer, nonsmall cell lung cancer, and glioma by diverse mechanisms such as genomic amplification, chromosomal translocations, disruption of normal intercellular trafficking, and proteolysis [22–25]. It is generally recognized that Cyclin D1 is associated with tumor malignancy and poor prognosis. Recently, suppression of Cyclin D1 reduced the proliferation and invasiveness of glioma cells while inducing apoptosis, suggesting the crucial role of Cyclin D1 in gliogenesis and defining Cyclin D1 as a promising molecular target for anticancer therapy [26]. In addition, an increasing number of reports have shown that Cyclin D1 was involved in the miRNAs and downstream target genes regulatory networks. MiR-9 suppresses the expression of Cyclin D1 via directly targeting 3′-UTR of Cyclin D1, inhibiting the proliferation, invasion, and metastasis of gastric cancer cell in vitro and in vivo [27]. Abnormal suppression of miR-503 leads to the increase in the Cyclin D1 level, which may promote carcinogenesis in endometrioid endometrial cancer [28]. miR-195 inhibits glioma cell proliferation by downregulating expression of Cyclin D1 and cyclin E1, through directly targeting the 3′-UTR of Cyclin D1 and cyclin E1 [29]. In the present study, we further revealed that the inhibiting effect of miR-15b on the proliferation and apoptosis of U87 and LN229 cell could be mediated by reducing the expression of Cyclin D1. Results from the luciferase reporter assay suggested that Cyclin D1 was one of the direct functional downstream targets of miR-15b. MiR-15b regulates Cyclin D1 expression through targeting the 3′ UTR of Cyclin D1, which was confirmed by western blotting analysis.

To conclude, our results show that miR-15b reduces in glioma specimens and cell lines, highlighting that miR-15b is a tumor suppressor miRNA in progression of glioma. Overexpression of miR-15b inhibits proliferation by arrested cell cycle progression and induces apoptosis, possibly by directly targeting Cyclin D1. Collectively, these data suggest that miR-15b and Cyclin D1 may be potential therapeutic targets for gliomagenesis and deserve further study.
